# Evaluation of the medicinal properties of *Cyrtocarpa procera* Kunth fruit extracts

**DOI:** 10.1186/s12906-015-0602-y

**Published:** 2015-03-21

**Authors:** Karla Stephanie Martinez-Elizalde, Manuel Jimenez-Estrada, Cesar Mateo Flores, Luis Barbo Hernandez, Rocio Rosas-Lopez, Angel Duran-Diaz, Oscar J Nieto-Yañez, Elizabeth Barbosa, Marco Aurelio Rodriguez-Monroy, Margarita Canales-Martinez

**Affiliations:** Laboratorio de Farmacognosia, Unidad de Biotecnologia y Prototipos (UBIPRO) Facultad de Estudios Superiores Iztacala, Universidad Nacional Autonoma de Mexico, Av. De los Barrios No.1, Los Reyes Iztacala, Edo. Mex, Tlalnepantla, C.P. 54090 Mexico; Laboratorio de Inmunobiología, Carrera de Medicina, Facultad de Estudios Superiores-Iztacala UNAM, Edo. Mex, Tlalnepantla, Mexico; Laboratorio de Fisiología Vegetal, UBIPRO Facultad de Estudios Superiores-Iztacala UNAM, Edo. Mex, Tlalnepantla, Mexico; Instituto de Quimica, UNAM, Distrito Federal, Mexico; Universidad de la Cañada, Teotitlan de Flores Magon, Oaxaca, Mexico; Escuela Superior de Medicina IPN, Distrito Federal, Mexico

**Keywords:** *Cyrtocarpa pocera*, *Anacardiaceae*, Medicinal fruit, Antibacterial activity

## Abstract

**Background:**

The fruit of *Cyrtocarpa procera* is used to treat stomach diseases by people living in San Rafael, Coxcatlan, Puebla. This work investigated the antibacterial, antioxidant, cytotoxic and anti-inflammatory activities of the fruit produced by this species.

**Methods:**

Methanol extract was obtained by maceration. After obtaining the methanol extract (MeOH1), methanol subextract (MeOH2) and hexane (H) were obtained. The antibacterial activities of MeOH1, MeOH2 and H were evaluated through disc-diffusion. The quenching of free radicals was evaluated by decolorizing a methanolic DPPH solution. The cytotoxic activity of MeOH2 was evaluated by *in vitro* assay system of growth inhibition of human cervical carcinoma cell line (CasKi). The IL-1β and TNF-α were determined through ELISA in the supernatants of the macrophage cell line (RAW 264.7). The MeOH2 subextract was separated by column chromatography, seventy-three fractions were collected.

**Results:**

The Gram-positive and -negative bacteria examined were sensitive to MeOH1 and MeOH2; the MeOH2 was bactericidal toward *Staphyloccocus aureus* (MIC = 4 mg/mL) and *Vibrio cholera* (MIC = 4 mg/mL). The MeOH2 inhibited the DPPH radical (SC_50_ = 69.7 μg/mL), but a cytotoxicity assay revealed that the extract is not toxic according to the National Cancer Institute (LD_50_ = 22.03 μg/mL). The production of proinflammatory cytokines (IL- 1β and TNF- α) by LPS- stimulated macrophages was reduced after the treatments. The methanol extract contained various organic acids, such as citric acid, palmitic acid and α- linoleic acid.

**Conclusions:**

The fruits of *Cyrtocarpa procera* are employed to treat ailments such as diarrhea, in this study were demonstrated some biological activities involved in a bacterial infection. This is the first research about of the medicinal properties of *C. procera* fruit.

## Background

Mexico displays enormous biotic diversity; this country has the fourth best diversity of phanerogams plants, containing approximately 30,000 species that represent between 10 and 12% of species worldwide [1].

*Cyrtocarpa procera* Kunth is an indigenous Mexican tree that belongs to the *Anacardiaceae* plant family. This species is used in traditional Mexican medicine, known locally as “chupandilla” or “copalcojote”, the bark and fruit are employed to treat ailments such as diarrhea, dysentery and cough [2-5]. This species is endemic to Mexico and is distributed in the center of the country (Colima, Michoacan, Estado de Mexico, Oaxaca, Morelos, Jalisco, Nayarit, Guerrero and Puebla states). *C. procera* is a species found in Tehuacan-Cuicatlan Valley (Puebla, Mexico); this area is critical for the conservation of one of the main biodiversity reserves among the arid and semi-arid areas of Mexico [5]. *C. procera* is one of the medicinal plants used by the inhabitants of San Rafael, a town located within the Tehuacan-Cuicatlan Valley; this location participates in a line of a regional ethnobotanical research focusing on the current condition of natural resources and providing the tools necessary for using and preserving the natural resources inside the reserved biosphere.

Several studies have described the phytochemical and medicinal properties of *C. procera* bark [4,6,7] however, the medicinal properties of the chupandilla fruit have not been assessed. Therefore, the purpose of the present study was to evaluate the medicinal properties of this fruit.

## Methods

### Plant material

The *C. procera* fruit was collected in August 2012 in San Rafael, Coxcatlan, Puebla and the botanical authentication of the specimen was done by M. C. Maria Edith Lopez Villafranco (curator at the IZTA Herbarium). Voucher specimens were deposited in the herbarium IZTA at the Facultad de Estudios Superiores Iztacala (voucher no. 2412 IZTA).

San Rafael is a village in the municipality of Coxcatlan, which is located southeast of the Tehuacan-Cuicatlan Valley at 18°12’ and 18°14’ North and 97°07’ and 97°09’ West, residing 957 m above sea level. The climate is dry or arid with summer rains and a mean temperature of 22°C [8].

The specimens were collected in the field with permission from the “Secretaria de Medio Ambiente y Recursos Naturales” (SGPA/DGVS/1266).

### Preparation of the extracts

The extract of the *Cyrtocarpa procera* fruit was obtained from dehydrated fruits (mature: 690.78 g) through maceration with methanol (2.0 L) at room temperature. After filtration, the solvent was evaporated under reduced pressure, generating the methanol extract (MeOH1). The yield of MeOH1 was 164.95 g (23.87%). Sixty g of the MeOH1 were dissolved in methanol (500 mL) and hexane (500 mL) before being placed in a separatory funnel. After the solvent–solvent extraction, the methanol subextract (MeOH2) was removed from the hexane (H). After removing the solvent, 53.58 g of MeOH2 (89.3%) and 4.28 g of H (7.13%) were obtained.

### Antibacterial activity

The following strains of bacteria were used: *Vibrio cholerae* (Instituto de Diagnóstico y Referencia Epidemiológicos INDRE 206,isolated from polluted water), *Vibrio cholerae* (a clinical isolate corresponding to group 01 that produces enterotoxin and has the serotype “Inaba” and the biotype “El Tor”), *Escherichia coli* (American Type Culture Collection, ATCC 25922), *Salmonella typhi* ATCC 19430, *Staphylococcus aureus* ATCC 12398, *Staphylococcus aureus* ATCC 29213, *Enterobacter aerogenes, Staphylococcus epidermidis, Bacillus subtilis, Enterococcus faecalis*, *Proteus mirabilis* (donated by the Laboratory of Microbiology of FES-Cuatitlan UNAM) and *Yersinia enterocolitica* (donated by the Clinical Analysis Laboratory of FES-Iztacala UNAM).

The antibacterial activity was measured through disc–diffusion [9]. The microorganisms were grown overnight at 37°C in 10 mL of Müeller Hinton broth (Bioxon 260–1, Estado de Mexico, Mexico). The cultures were adjusted to turbidities comparable to that of a Mc Farland no. 0.5 standard with sterile saline solution. Petri dishes containing Müeller Hinton agar (Bioxon, Edo. de Mexico, Mexico) were impregnated with these microbial suspensions. Subsequently, 200 mg/mL solutions of each extract were prepared, and 5-mm discs (Whatman no. 5) were impregnated with 10 μL of each extract (final doses per disc: 2 mg of MEOH1, MEOH2 and H). Discs impregnated with 10 μL of hexane and methanol were used as negative controls. Discs containing chloramphenicol (25 μg) were used as positive controls. Plates were incubated overnight at 37°C, and the diameter of any resulting inhibition zones (mm) was measured. Each experiment was repeated at least three times. The minimal inhibitory concentration (MIC) was estimated using the broth dilution method [9]. Diluted plant extracts (10.0 to 0.125 mg/mL) were used. Tubes were inoculated with a 1×10^5^ CFU/mL microorganism suspension. MIC values were defined as the lowest extract concentration that prevents visible bacterial growth after 24 h of incubation at 37°C. Each experiment was repeated at least three times. The bactericidal kinetic assay was performed using the appropriate concentrations of the extract (corresponding to MIC_50_, MIC and MBC) [10].

### DPPH decoloration assay

The ability of the extracts to quench free radicals was evaluated spectrophotometrically at 517 nm by decolorizing a methanolic 2,2-Diphenyl-1-picryl-hydrazyl (DPPH) solution, as described by Murillo [11]. A freshly prepared DPPH solution (4 mg/100 mL methanol) was used during the assays. Samples were dissolved in methanol (1–100 μg/mL), and the DPPH solution was a control. The degree of decoloration reveals the free radical scavenging efficiency of the samples. Quercetin was used as a reference free radical scavenger (SC_50_ = 4.6 μg/mL). The percentage of DPPH decoloration was calculated as follows:

$$ \mathrm{Inhibition}\ \mathrm{p}\mathrm{ercentage}\ \left(\mathrm{I}\mathrm{p}\right) = \left[\left({\mathrm{A}}_{\mathrm{B}}\hbox{--}\ {\mathrm{A}}_{\mathrm{A}}\right)/\ {\mathrm{A}}_{\mathrm{B}}\right)\Big] \times 100 $$

where A_A_ is the absorbance of the sample, and A_B_ is the absorbance of the control [12].

The SC_50_ values were calculated through rectangular hyperbola regressions, where the abscissa represented the concentration of the tested plant extract, and the ordinate represented the average percentage of the scavenging capacity from three replicates.

### Cytotoxic activity

A human cervical carcinoma cell line (CasKi) was purchased from the American Tissue Culture Collection (ATCC, USA). Cells were maintained in RPMI 1640 medium (Sigma Aldrich, St. Louis, MO, USA) supplemented with 10% fetal bovine serum (GIBCO, The Grand Island, New York, USA), 100 μg/mL gentamycin (GIBCO) and 50 μg/mL of fungizone (GIBCO). Cells were cultured under a humidified atmosphere containing 5% CO_2_ in an incubator kept at 37°C.

### *In vitro* crystal violet cytotoxicity assay

Briefly, the cells (3×10^4^/well) were seeded in 96-well plates and allowed to grow for 24 hours before treatment. Afterwards, cells were treated with 11 different concentrations (250 – 0.24 μg/mL) of MeOH2 in three replicates. The plates were incubated for 72 h at 37°C under 5% CO_2_. A stock solution was initially obtained by dissolving the MeOH2 in DMSO (Sigma). The different concentrations were achieved through dilution while ensuring that the final concentration of DMSO in the test and control wells did not exceed 1% (v/v). DMSO did not induce an observable effect. Doxorubicin was used as the positive control. Wells containing untreated cells was the negative control. At the end of the incubation period, the viability was evaluated through a dye uptake assay performed according to Badisa et al. [13]. Glutaraldehyde (30 μL at 1.1%) was added to each well and incubated for 30 min at room temperature to fix the cells. The plates were rinsed with PBS 10X to wash off the dead cells and dried under flowing air inside a laminar hood for 5 to 10 min. Crystal violet (SIGMA, St. Louis, MO, USA) (50 μL of 0.1%) was added to each well, and the samples were incubated for 15 min, washed and dried. To solubilize the dye, 50 μL of 10% acetic acid were added to each well, and the plates were read at 570 nm in a Bio-Tek EL800 plate reader (Bio-Tek, Winooski, VT, USA).

The cytotoxicity of each sample is expressed as a IC_50_ value. The IC_50_ value is the concentration of test compounds that cause 50% inhibition or cell death; this value was the average of three experiments and was obtained by plotting the percentage inhibition versus the concentration of MeOH2. According to the NCI (National Cancer Institute) plant screening program, a plant extract is generally considered to be actively cytotoxic if the IC_50_ value is 20 μg/mL or less after incubation for 48 to 72 hours; this threshold is 4 μg/mL or less for pure compounds [14].

### *In vitro* determination of cytokine production

The macrophage cell line RAW 264.7 was obtained from the American Tissue Culture Collection (ATCC, USA). Cells were maintained in DMEM (Sigma) supplemented with fetal bovine serum 10% (GIBCO), 100 μg/mL gentamicin (GIBCO) and 50 μg/mL fungizone (GIBCO), the cells were cultured in a humidified atmosphere containing 5% CO_2_ at 37°C. Cells were detached using 0.5% Trypsin-Versene.

The cells were seeded in a 24-well culture plate at 1×10^6^ cells per well and allowed to incubate for 2 hours before treatment. Afterwards, cells were washed with PBS 1X and treated with different concentrations of the fruit extract (MeOH1, MeOH2 and H) (7.43, 11 and 4.56 μg/mL, respectively) from *C. procera* in triplicate. The plates were incubated for 24 h under 5% CO_2_ at 37°C, and the culture medium was recovered in 1.5 mL microcentrifuge tubes before being frozen at −20°C.

The IL-1β and TNF-α were determined through ELISA in the supernatants of the macrophage cell line. To prepare the cell supernatants, the macrophages were washed twice, adjusted to 10^6^ cells/mL, and cultured at 37°C in 5% CO_2_ for 24 h in complete RPMI medium either alone or with 1 μg/mL LPS with and without increasing doses of MeOH2. After 24 h, the supernatants were collected by centrifugation at 1000 *g* for 20 min at 18°C and assayed for IL - 1β (range of detection 63 to 4000 pg/mL) and TNF-α (range of detection 16 to 2000 pg/mL) using ELISA kits according to the manufacturer’s instructions (Peprotech).

### Fractionation of MeOH2

The MeOH2 was subjected to silica gel column chromatography (silica gel mesh 70–230SIGMA 5–2509, St. Louis, MO, USA; 40 cm long, 5.5 cm internal diameter). The column was eluted with the following gradient of solvents hexane:ethyl acetate, ethyl acetate, ethyl acetate:methanol. Seventy-three fractions were collected. The active compounds were purified through assay-guided isolation; the antibacterial activity of the collected partitions was measured through disc–diffusion [9]. The most active fractions, MeOH2 and H were analyzed with an AGILENT 6850 (China) gas chromatograph equipped with a HP-5MS (USA) column (30 m × 0.25 mm i.d., film thickness 0.25 μm). The temperature of the column was programmed starting at 70°C for 2 min, and the temperature was increased at 8°C/min up to 270°C. Then, at 270°C, a linear gradient was programmed to increase the temperature at 10°C/min up to 290°C. The injector and detector temperatures were 250 and 290°C, respectively. The carrier gas was helium at 0.9 mL/min. The peak areas were measured through electronic integration. The relative amounts of the individual components were based on the peak areas. The GC-MS analysis was performed on an AGILENT 5975C (China) mass spectrometer. The mass spectra were recorded at 70 eV. The partitioned components were identified by comparing their retention indices and mass spectra with data in the NIST/EPA/NIH Mass Spectral Library.

### Statistical analysis

All experiments were performed in triplicate. The means and standard deviations of the three experiments were determined. The IC_50_ and SC_50_ values were calculated through a rectangular hyperbola model. The statistical differences between the treated and control groups were evaluated through an analysis of variance (ANOVA). Values of P < 0.05 (*) were considered to be statistically significant.

## Results and discussion

The fruit of *C. procera*, which is also known as Chupandilla, is used as food and medicine with antidiarrheal activity [2]. The antibacterial, antioxidant, cytotoxic and anti-inflammatory activities are reported for the first time in the present study.

The results obtained when evaluating the antibacterial activity of the extract and subextract of *Cyrtocarpa procera* fruit are shown in Table 1. H was not active. MeOH1 and MeOH2 were active against the Gram-positive and Gram-negative bacteria tested. Significant differences between the antibacterial activity of the MeOH1 and MeOH2 were evident, MeOH2 exhibited the strongest antibacterial activity. The MeOH1 was active against three Gram-positive bacteria (two strains of *S. aureus* and *B. subtillis*) and six Gram-negative bacteria (two strains of *V. cholerae, E. aerogenes, E. coli,* one strain of *Y. enterocolitica* and *P. mirabilis)*, and this extract exhibited the lowest MIC in *S. aureus* and *V. cholerae* Tor (MIC = 4 mg/mL). The MeOH2 was active against five Gram-positive bacteria (two strains of *S. aureus, S. epidermidis, B. subtillis* and *E. faecalis*) and six Gram-negative bacteria (two strains of *V. cholerae, E. aerogenes, E. coli,* one strain of *Y. enterocolitica* and *P. miriabilis*); this extract exhibited the lowest MIC in *S. aureus* and *V. cholerae* Tor (MIC = 2 and 4 mg/mL, respectively).

**Table 1:** **Antimicrobial activities of the** ***Cyrtocarpa procera*** **fruit**

**Bacteria**	**Positive control**	**MeOH1**	**CMI**	**MeOH2**	**CMI**
	**Chloramphenicol**	**(mm)**	**(mg/mL)**	**(mm)**	**(mg/mL)**
Sa 29213	10.00 ± 1.00	7.00 ± 0.00	4.00	8.00 ± 0.00	2.0
Sa 12398	28.00 ± 0.00	9.00 ± 0.00	5.00	9.00 ± 0.00	2.0
Se	30.30 ± 0.57	Na	Na	9.00 ± 0.00	6.0
Bs	24.00 ± 1.00	9.00 ± 0.00	2.00	10.00 ± 0.00	4.0
Ef	24.30 ± 0.57	Na	Na	8.33 ± 0.58	6.0
Vch Indre	8.33 ± 0.58	7.67 ± 0.58	4.00	9.00 ± 0.00	5.0
Vch Tor	7.33 ± 0.58	8.00 ± 0.00	4.00	9.33 ± 0.58	4.0
Ea	22.00 ± 0.00	7.00 ± 0.00	5.00	8.00 ± 0.00	6.0
Ec	21.67 ± 1.70	7.67 ± 0.58	8.00	7.00 ± 0.00	10.0
St	28.00 ± 1.63	Na	Na	Na	Na
Ye CUSI	25.67 ± 0.47	7.00 ± 0.00	4.00	8.33 ± 0.58	6.0
Pm	17.30 ± 0.57	Na	Na	9.33 ± 0.58	>10.0

Sa 29213: *Staphylococcus aureus* ATCC 29213; Sa 12398: *S. aureus* ATCC 12398; Se: *S. epidermidis*; Bs: *Bacillus subtilis*; Ef: *Enterococcus faecalis;* Vch Indre, *Vibrio cholera* (isolated from water); Vch Tor: *Vibrio cholera* CDC V12; Ea: *Enterobacter aerogenes;* Ec: *Escherichia coli*; St: *Salmonella typhi*; Ye CUSI: *Y. enterocolitica* CUSI*;* Pm, *Proteus mirabilis.* Na: no activity.

**Figure 1:**
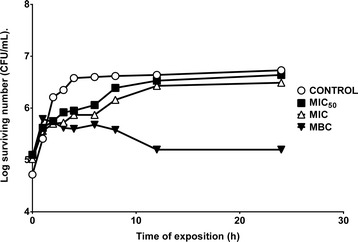
**Survival curve for** ***Staphylococcus aureus*** **29213 exposed to MeOH2 of C** ***yrtocarpa procera*** **.** The **MeOH2** was added to each experimental culture at time zero. The concentrations were 1.00 mg/mL (MIC_50_), 4.00 mg/mL (MIC) and 8.00 mg/mL (CBM); the control tube did not contain **MeOH2**.

**Figure 2:**
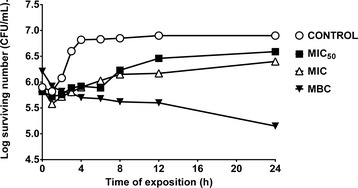
**Survival curve for the** ***Vibrio cholerae*** **Tor exposed to MeOH2 from** ***Cyrtocarpa procera.*** The **MeOH2** was added to each experimental culture at time zero. The concentrations were 2.00 mg/mL (MIC_50_), 4.00 mg/mL (MIC) and 8.00 mg/mL (MBC); the control tube did not contain **MeOH2**.

Figures 1 and 2 show the effects of the MeOH2 on the survival curves against *S. aureus* and *V. cholerae* Tor. The minimum bactericidal concentration (MBC = 8 mg/mL) had a bacteriostatic effect, similar activity is shown against *V. cholerae* Tor (MIC = 8 mg/mL). Most gastrointestinal diseases are associated with these bacterial groups [15-17], which is why we evaluated the extracts against these types of bacteria. Furthermore, other fruits from the Anacardiaceae family with antibacterial activity have been evaluated and tested. *Rhus typhina* (Anacardiaceae), originated in North America, is used to make a beverage termed “sumac-ade” or “rhus juice” prepared from its fruits and serves also as a traditional medicine. The antibacterial activity was determined and Gram-positive bacteria were generally found to be more sensitive than Gram-negative bacteria [18]. A similar observation was made on another species of the Anacardiaceae family, *Mangifera indica* seed kernel extracts [19] and on *Schinus molle* fruit essential oil [20].

The MeOH2 exhibited the highest antioxidant activity, efficiently scavenging the DPPH free radical with a SC_50_ value of 69.7 μg/mL, followed by MeOH1 (SC_50_ = 80.6 μg/mL) and H (90.1 μg/mL). One of the early responses of host innate immunity is Reactive Oxygen Species (ROS) production against microbial invaders. Free oxygen radicals are highly toxic toward pathogens and are utilized to prevent colonization by microorganisms in tissues, facilitating pathogen clearance and contributing to the signaling cascades related to inflammation [21], for this reason we evaluated the antioxidant activity. This property has been assessed in other fruits from Anacardiaceae family, and the SC_50_ values were both higher and lower than those we obtained [18,22].

**Figure 3:**
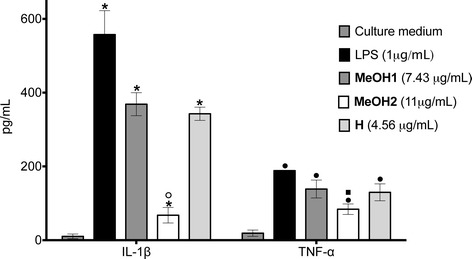
**Effect of the** ***Cyrtocarpa procera*** **fruit extracts on the LPS-induced cytokine secretion.** The macrophages RAW264.7 (1 × 10^6^ cells/well) were plated in 24-well plates and stimulated with LPS (1 μg/mL) in the absence or presence of the extracts at the indicated concentrations for 24 h. Afterwards, culture medium was collected to determine the IL-1β, TNF-α by ELISA kits. The results are expressed as the mean ± SEM of cytokine concentration (μg/mL). (**p < 0.01) versus LPS-stimulated cells.

MeOH2 significantly reduces the production of pro-inflammatory cytokines, such as IL-1β and TNF- α, by macrophages (Figure 3). MeOH2 decreased the production of proinflammatory cytokines, such as IL-1β and TNF-α, most likely because the extract containsα-linoleic acid. TNF-α is the major regulatory cytokine for inflammation. A number studies reported that conjugated linoleic acid can reduce TNF-α levels. This suggests that the observed dietary effects of conjugated linoleic acid may occur directly at the level of the macrophages/monocytes which are responsible for producing the majority of inflammatory cytokines [23,24].

MeOH2 shows no cytotoxic activity against CasKi cells (IC_50_ = 22.03 μg/mL), which is why this fruit is edible; the MeOH1 and H (IC_50_ = 14.86 and 9.12 μg/mL, respectively) were toxic, and they have anti-cancer activity. According to the National Cancer Institute (NCI), extracts are considered active at ≤ 20 μg/mL, while pure compounds are considered active at ≤ 4 μg/mL [14]. Other studies have revealed cytotoxic activity in the medicinal fruits of the Anacardiaceae family [25]; nevertheless, several reports indicate that the fruit extracts did not have cytotoxic activity [26]. However, we recognized that whether an IC_50_ value corresponds to a significant or non-significant cytotoxicity depends on the sensitivity of the cell line. Cancer cells are normally highly-specialized cells which have regressed to a much simpler, more primitive stage and which, unlike the normal parent, divide continuously, although inefficiently. Because a much higher proportion of cancer cells are undergoing active division, they are more vulnerable than most normal cells to anti-cancer drugs. However, normal tissues with high mitotic indices (e.g. bone marrow, spleen, thymus and intestinal epithelium) are also more susceptible to anti-cancer drugs [27].

From MeOH2, we obtained seventy-three fractions from a silica gel column; only fractions 4, 9, 14, 15, 18 and 24 were active against *V. cholerae* Tor, and fraction 4 exhibited a larger inhibition halo (13 mm).

The GC-MS analysis of MeOH2, H and fractions 4, 9, 14, 15, 18 and 24 revealed different compounds (Table 2). These compounds have various biological activities. Itaconic acid, 1,2-benzenedicarboxylic acid, mono(2-ethylhexyl) ester, coumaran, fatty acids, tetradecane inhibit the growth of bacteria [28-31]. Palmitic, linoleic and stearic acids are known to have potential antibacterial and antifungal agents. Fatty acids are known for their antimicrobial action, particularly against Gram positive bacteria. The observed inhibition is explained as a consequence of the uptake of undissociated fatty acids which dissipate the transmembrane proton gradient and thereby affect ATPase activity [32]. The undissociated form of fatty acids is highly soluble in membrane phospholipids and has been shown to enter the cell by passive diffusion [33]. Additionally, long-chain unsaturated fatty acids are bactericidal to important pathogenic microorganisms, including Methicillin-resistant *Staphylococcus aureus*. These antibacterial actions of fatty acids are usually attributed to long-chain unsaturated fatty acids including linoleic acid, while long-chain saturated fatty acids, including palmitic acid and stearic acid, are less active [34,35]. Linoleic acid exhibited inhibition of *S. aureus* FabI, but palmitic acid and stearic acid did not inhibit Fab I activity. Fab I has been identified as a target for antibacterial drug development [36]. Itaconic acid is another organic compound with antibacterial activity because it inhibits isocitrate lyase, the key enzyme of the glyoxylate shunt, a pathway essential for bacterial growth under specific conditions [29].

**Table 2:** **GC-MS data for the MeOH2, hexane and MeOH2 fractions**

	**Retention Time (min)**	**Compound**	**Abundance (%)**
MeOH2	20.48	Palmitic acid	0.53
	22.62	α-linoleic acid	0.86%
	22.78	Stearic acid	53.66
	28.77	Quinone	1.71
Hexane	20.09	Palmitic acid	22.26
	22.14	α-linoleic acid	40.36
	22.48	Stearic acid	2.20
	32.84	Vitamin E	5.83
4	11.902	Citric acid	23.0
	17.258	Palmitic acid	3.89
	22.506	1,2-Benzenedicarboxylic acid, mono(2-ethylhexyl) ester	27.53
9	8.48	Coumaran	9.04
	8.97	Tetradecane	17.73
	11.895	Citric acid	3.79
14	6.66	Itaconic acid, dimethyl ester	20.83
	11.895	Citric acid	32.43
15	4.29	2H-Pyran-2-one	10.86
	6.67	Itaconic acid, dimethyl ester	7.73
	11.902	Citric acid	22.64
	4.126	2H-Pyran-2-one	60.21
18	11.876	Citric acid	5.35
24	4.12	Itaconic anhydride	39.37
	10.477	Citric acid	3.67

MeOH2 and H: subextract; 4, 9, 14, 15, 18, 24: the most active fractions of the column chromatography.

Moreover some compounds have antioxidant and anti-inflammatory activities (coumaran, benzenedicarboxylic acid, mono(2-ethylhexyl) ester, palmitic acid, linoleic acid [31,37]. DPPH is a free radical compound and has been widely used to test the free radical-scavenging ability of various samples. It was clear that the presence of antioxidant compounds in the MeOH2 showed free radical scavenging activity on DPPH. Antioxidants, upon interaction with DPPH, either transfer an electron or a hydrogen atom to DPPH, thus neutralizing its free radical character [38]. Spices and herbs, used in foods and in medicinal mixtures contain compounds that have strong H-donating activity [39]. For exemple, the oxidation of linoleic acid (due to the abstraction of a hydrogen atom from diallylic methylene groups) and the reduction of DPPH [40].

## Conclusion

The fruits of *Cyrtocarpa procera* are employed to treat ailments such as diarrhea. In this study were demonstrated the antibacterial, antioxidant, anti-inflammatory and no cytotoxic activities of MeOH2 subextract. The compounds identified by the phytochemical analysis explained the biological activities. This is the first research about of the medicinal properties of *C. procera* fruit.
